# Mitochondrial Calcium Uniporter (MCU) is Involved in an Ischemic Postconditioning Effect Against Ischemic Reperfusion Brain Injury in Mice

**DOI:** 10.1007/s10571-024-01464-7

**Published:** 2024-04-03

**Authors:** Hiromitsu Sasaki, Ichiro Nakagawa, Takanori Furuta, Shohei Yokoyama, Yudai Morisaki, Yasuhiko Saito, Hiroyuki Nakase

**Affiliations:** 1 Department of Neurosurgery, Nara Medical University, Shijo-Cho 840, Kashihara City, Nara 634-8522 Japan; 2 Department of Neurophysiology, Nara Medical University, Shijo-Cho 840, Kashihara City, Nara 634-8522 Japan

**Keywords:** Ischemic postconditioning (PostC), Mitochondrial calcium uniporter (MCU), NMDA receptor (NMDAR), Mitochondrial permeability transition pore (mPTP)

## Abstract

The phenomenon of ischemic postconditioning (PostC) is known to be neuroprotective against ischemic reperfusion (I/R) injury. One of the key processes in PostC is the opening of the mitochondrial ATP-dependent potassium (mito-K_ATP_) channel and depolarization of the mitochondrial membrane, triggering the release of calcium ions from mitochondria through low-conductance opening of the mitochondrial permeability transition pore. Mitochondrial calcium uniporter (MCU) is known as a highly sensitive transporter for the uptake of Ca^2+^ present on the inner mitochondrial membrane. The MCU has attracted attention as a new target for treatment in diseases, such as neurodegenerative diseases, cancer, and ischemic stroke. We considered that the MCU may be involved in PostC and trigger its mechanisms. This research used the whole-cell patch-clamp technique on hippocampal CA1 pyramidal cells from C57BL mice and measured changes in spontaneous excitatory post-synaptic currents (sEPSCs), intracellular Ca^2+^ concentration, mitochondrial membrane potential, and *N*-methyl-d-aspartate receptor (NMDAR) currents under inhibition of MCU by ruthenium red 265 (Ru265) in PostC. Inhibition of MCU increased the occurrence of sEPSCs (*p* = 0.014), NMDAR currents (*p* < 0.001), intracellular Ca^2+^ concentration (*p* < 0.001), and dead cells (*p* < 0.001) significantly after reperfusion, reflecting removal of the neuroprotective effects in PostC. Moreover, mitochondrial depolarization in PostC with Ru265 was weakened, compared to PostC (*p* = 0.004). These results suggest that MCU affects mitochondrial depolarization in PostC to suppress NMDAR over-activation and prevent elevation of intracellular Ca^2+^ concentrations against I/R injury.

**Graphical Abstract:**

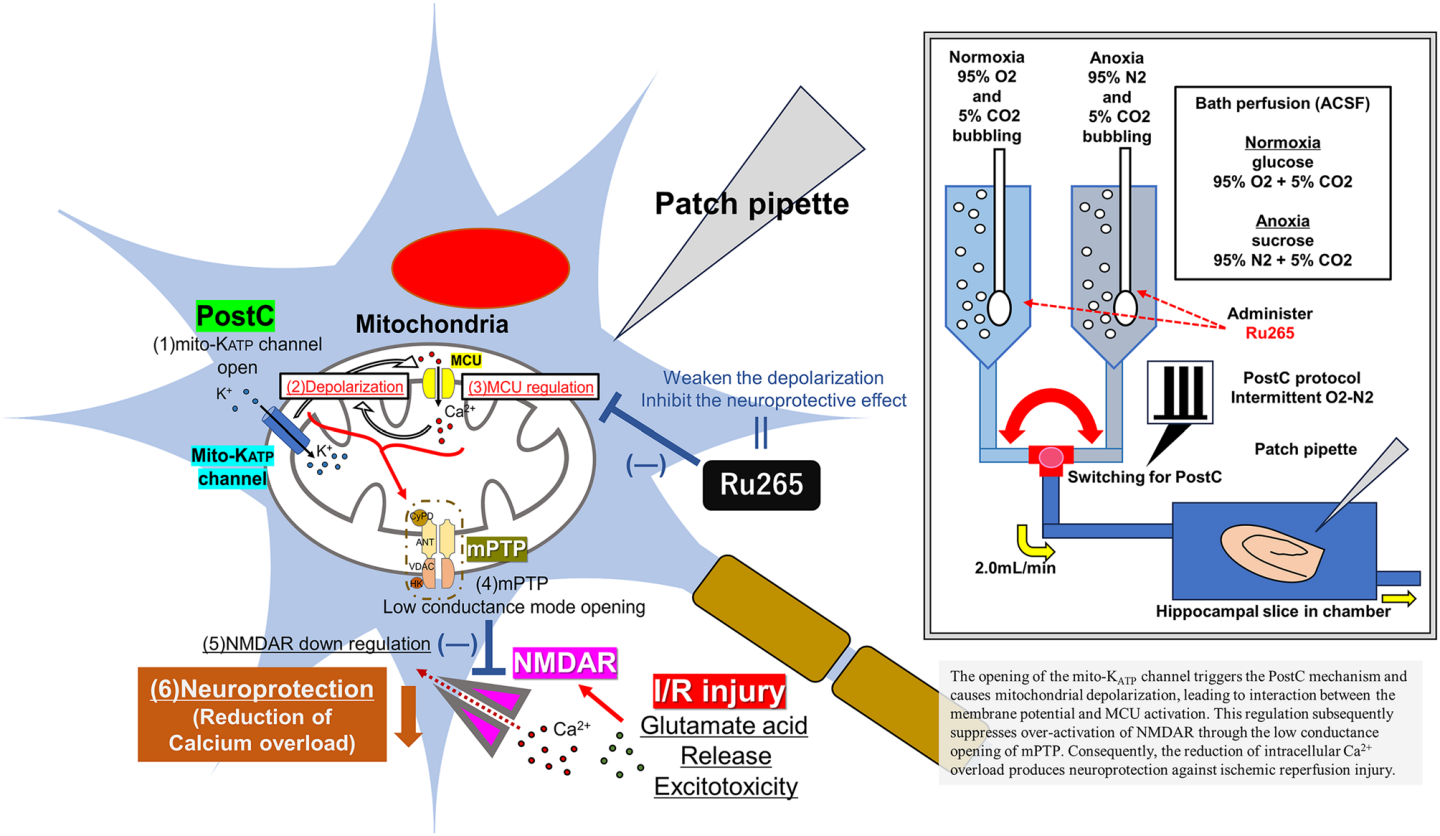

**Supplementary Information:**

The online version contains supplementary material available at 10.1007/s10571-024-01464-7.

## Introduction

Cerebral ischemic reperfusion (I/R) injury is a common characteristic of ischemic stroke and occurs when the restoration of blood flow after a period of ischemia results in damage to neurons. The recovery of function in neurons damaged by ischemia could thus be limited, even though reperfusion represents the main treatment for acute ischemic stroke (AIS). Conversely, the phenomenon leading to the acquisition of ischemic tolerance called “ischemic preconditioning” is known to provide marked neuroprotective effects against I/R injury, triggered by placing mild intermittent ischemic loads on the brain prior to fatal ischemic assault (Kitagawa et al. 1990; Nakagawa et al. 2002; Yin et al. 2005). However, predicting when AIS will occur is very difficult in clinical situations and provision of ischemic preconditioning for patients before AIS onset is not feasible. However, even after a severe ischemic assault has occurred, the provision of a mild, intermittent ischemic load can exert a neuroprotective effect called ischemic postconditioning (PostC) (Pignataro et al. 2009; Xing et al. 2008).

This concept of PostC appears applicable as a new therapeutic approach to AIS along with intravenous administration of tissue-plasminogen activator and mechanical thrombectomy. One of the key processes in I/R injury is reportedly the over-activation of N-methyl-D-aspartate receptor (NMDAR), which induces an excessive influx of Ca^2+^ into cytoplasm via the opening of NMDAR, leading to cytoplasmic Ca^2+^ overload and activation of various proteins such as caspases and endonucleases via high-conductance opening of the mitochondrial permeability transition pore (mPTP) (Hawrysh and Buck 2013). This mechanism of I/R injury is also the key to PostC phenomenon because our previous studies have shown that PostC is triggered through the opening of mitochondrial adenosine triphosphate (ATP)-dependent potassium (mito-KATP) channels and suppresses this over-activation of NMDAR (Morisaki et al. 2022; Yokoyama et al. 2019). In more detail, we revealed that opening of the mito-KATP channel caused depolarization of the mitochondrial membrane and induces reductions in NMDAR currents through low-conductance opening of mPTP. As a consequence, Ca^2+^ influx into cytoplasm is suppressed, leading to neuroprotection against I/R injury (Morisaki et al. 2022; Yokoyama et al. 2019).

Elucidation of the detailed mechanisms of PostC is expected to yield beneficial information facilitating new treatments for AIS. However, it is unclear that the involvement between the mitochondrial Ca^2+^ metabolism and the PostC mechanism. Mitochondrial Ca^2+^ trafficking also plays key roles in many bioenergetic processes, such as mitochondrial respiration and the production of ATP, as the regulation of cytoplasmic Ca^2+^ concentrations is also essential for I/R injury and PostC. In this research, we focused on the mitochondrial calcium uniporter (MCU), a highly sensitive channel for the uptake of Ca^2+^ on the inner mitochondrial membrane that regulates mitochondrial Ca^2+^ concentrations (Clapham 2007; de Stefani et al. 2011). Many recent studies have reported structural data for the MCU, which comprises the following subunits: mitochondrial calcium uptake (MICU)1, MICU2, MICU3, MCUb, mitochondrial calcium uniporter regulator 1, and essential MCU regulatory element (Baughman et al. 2011; Mallilankaraman et al. 2012; Perocchi et al. 2010; Plovanich et al. 2013; Raffaello et al. 2013; Sancak et al. 2013; de Stefani et al. 2011). The subunits regulate one another to promote or suppress Ca^2+^ transport into the mitochondrion (Lambert et al. 2019; Patron et al. 2014). Numerous pathological conditions of cell activity, such as ischemic stroke, neurodegenerative disease, and cancer, are caused by the dysregulation of Ca^2+^ uptake by the MCU (Pchitskaya et al. 2018; Polster et al. 2017; Vultur et al. 2018; Woods and Wilson 2019), which has been thus attracting attention as a potential target of treatment in recent years (Giorgi et al. 2012; Liao et al. 2017; Modesti et al. 2021).

To the best of our knowledge, few reports have investigated the involvement of the MCU in PostC. We thus hypothesized that the MCU plays a role as a driving trigger of PostC and investigated relationships between PostC and the MCU by examining: (1) whether the MCU is involved in the mechanisms of PostC; (2) how Ca^2+^ kinetics involving the MCU are involved in the process; (3) whether downregulation of NMDAR currents occurs in PostC without the MCU; and (4) how mitochondrial membrane depolarization differs between PostC with MCU and PostC without MCU. We analyzed changes in sEPSCs, NMDAR current, cytosolic Ca^2+^ concentrations, and mitochondrial membrane potential under inhibition of the MCU by Ru265 and clarified the relationship between PostC and the MCU in hippocampal pyramidal neurons using a whole-cell patch-clamp technique.

## Materials and Methods

### Procedure for Obtaining Mouse Hippocampal Slices

All experiments were approved by the Animal Care and Use Committee of our University (Approval no. 13411) and performed all procedures in accordance with the Guidelines for the Proper Conduct of Animal Experiments. Four- to 8-week-old wild-type C57BL/6J mice (58 males) weighing about 18–24 g were used for our experiments. Mice were accommodated in cages with a 12-h:12-h light/dark cycle with ad libitum access to food and water. Mice were decapitated after we checked for the disappearance of the postural reflex and a change from shallow, fast breathing to deep, slow breathing under general anesthesia induced by isoflurane inhalation (0.05 *v/v*). Brains were removed and quickly transferred to an ice-cold solution of sucrose 230 mM, KCl 2.5 mM, NaHCO_3_ 25 mM, NaH_2_PO_4_ 1.25 mM, CaCl_2_ 0.5 mM, MgSO_4_ 10 mM, and d-glucose 10 mM bubbled with 95% O_2_/5% CO_2_. In this solution, brains were cut into horizontal slices of the hippocampus at a thickness of 350 µm, using a linear slicer (PRO7; DOSAKA EM, Kyoto, Japan). Slices were incubated in standard artificial cerebrospinal fluid (ACSF) comprising NaCl 125 mM, KCl 2.5 mM, NaHCO_3_ 25 mM, NaH_2_PO_4_ 1.25 mM, CaCl_2_ 2.0 mM, MgCl_2_ 1.0 mM, and D-glucose 10 mM bubbled with the same gas mixture and kept for at least 1 h at 32 °C. After this procedure, slices were kept in ACSF at 27 °C. We used about 5–7 slices per mouse.

### Perfusion Protocols and Patch-Clamp Recording

**Fig. 1:**
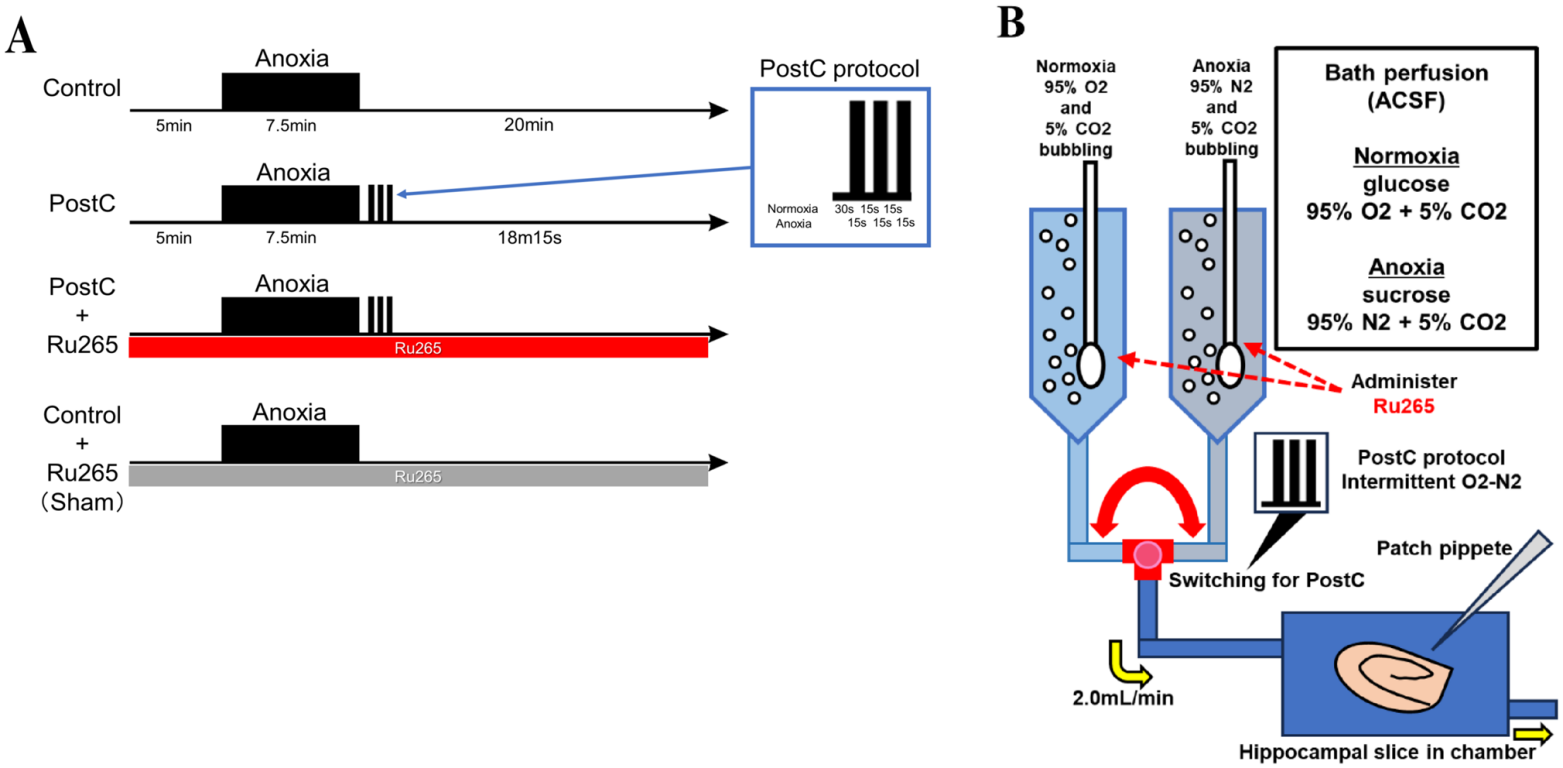
Anoxia-reperfusion protocols and patch-clamp technique. **A** The horizontal arrow indicates the time course. Black bands indicate anoxia and the red band indicates Ru265 perfusion in ACSF. All data were recorded for 32.5 min. In the PostC protocol, three cycles of 15 s of anoxia and 15 s of normoxia were provided. **B** Schema for patch-clamp recording. Each patch-clamp record was performed on the chamber while switching the flow of normoxic and anoxic ACSF with or without administration of Ru265. *PostC* ischemic postconditioning, *Ru265* ruthenium red 265; sham, control + Ru265 10 µM

Patch pipettes made from thick-walled borosilicate glass capillaries were filled with internal solution comprising Cs-gluconate (141 mM), CsCl (4.0 mM), MgCl_2_ (2.0 mM), HEPES (10.0 mM), Mg-ATP (2.0 mM), Na-GTP (0.3 mM), and EGTA (0.2 mM) adjusted to pH 7.25 with CsOH. Each slice was placed on an 800-µL recording chamber mounted on a BX50WI upright microscope (Olympus, Tokyo, Japan) that was equipped with an infrared differential interference microscope and epifluorescence imaging apparatuses. In this chamber, gas-saturated ACSF was perfused at 2.0 mL/min and kept at 31–33 °C by a controlled heater. Hippocampal slices were placed into this chamber and randomly assigned to the following three groups (Fig. 1A): (1) control group, comprising slices reperfused with ACSF for 20 min after the anoxic period; (2) PostC group, exposed after the anoxic period to intermittent normoxic and anoxic periods based on the PostC protocol described below; (3) PostC + Ru265 group that underwent the PostC protocol and were administered 1 µM, 10 µM, or 50 µM of ruthenium red 265 (Ru265) as a novel selective inhibitor of the MCU; and (4) Sham group that underwent the control protocol and were administered 10-µM Ru265. In the control group, the baseline time schedule included 5 min of normoxia, 7.5 min of anoxia, and 20 min of reperfusion. Severe anoxic load was simulated by exposing slices for 7.5-min to ACSF in which glucose and oxygen had been replaced with sucrose and nitrogen, respectively. In accordance with previous studies, the protocol for PostC was started from 30 s after the anoxic period and comprised three cycles of 15 s of anoxia and 15 s of normoxia (Yokoyama et al. 2019). In the PostC + Ru265 and sham groups, ACSF containing Ru265 was perfused from the start of the time schedule (Fig. 1A,B) . We then visually confirmed CA1 pyramidal cells and made whole-cell voltage-clamp recordings using an EPC-9 patch-clamp amplifier (HEKA Elektronik, Lambrecht/Pfalz, Germany). The holding potential of the voltage clamp was set at − 70 mV and resistance in the pipette was kept at 2.5–3.5 MΩ. To allow clear detection of glutamatergic excitatory post-synaptic currents, the GABA A and GABAB antagonist picrotoxin (50 µM) were added to ACSF during recordings. We counted the number of sEPSCs in each group, calculated as a percentage of the number of sEPSCs occurring during the anoxic and reperfusion periods compared to that in the first normoxic period for 5 min before the anoxic period. In this experiment, the three different concentrations of Ru265 (1 µM, 10 µM, or 50 µM) were applied to determine the optimal concentration of Ru265. Ru265 and picrotoxin were purchased from Sigma-Aldrich.

### Recording of Whole-Cell Currents in Response to NMDA Application

To assess NMDAR currents in each group, we recorded whole-cell currents after NMDA application. With another micropipette similar to that used for whole-cell recordings, 5 µM of NMDA was puffed onto the recording cell. The holding potential of the voltage clamp was set at − 55 mV during the pre- (1 s) and post-stimulation period (6 s) to suppress the Mg^2+^ blockade of NMDAR channels. All NMDAR currents were recorded every 30 s during experiments for about 30–35 min.

### Cell Staining

To investigate the effects of Ru265 in PostC, we counted the number of dead cells in hippocampal slices after ischemic injury. Dead cells were dyed by propidium iodide (PI) and SYTOX-blue, which cannot permeate cell membranes and are used for nuclear staining. At least 45 min before starting the perfusion protocol, slices were incubated for 15 min in ACSF containing 3-µM PI. As a result, PI staining was performed after slice preparation until the perfusion protocol started; that is, dead cells in which membranes had broken during the process of making slices were stained red. Subsequently, PI was removed before the perfusion protocol started, to avoid dyeing cells that died due to I/R injury during the perfusion protocol. According to the perfusion protocol, slices in the control group were loaded with the anoxic period for 7.5 min and the reperfusion period for 20 min, and slices in the PostC group and PostC + Ru265 group were loaded with intermittent anoxic periods after 7.5 min of anoxia. After these perfusion protocols, slices were transferred to the incubation chamber and stained with 6 µM of SYTOX-blue in ACSF for 3 h at 32 °C. In this procedure staining cells with SYTOX-blue, cells that were already dead were stained red with PI and cells that died in the perfusion protocol were stained blue. Cells that died in the slice preparation procedure were thus dyed both red and blue, while cells that died due to I/R injury were dyed blue only. SYTOX-blue was excited at 408 nm and the blue fluorescence emission was bandpass filtered from 417 to 477 nm using confocal microscopy (C2plus; Nikon, Japan). To detect the initial number of dead cells before ischemic stress, we also checked for dead cells stained with PI, which were excited at 561 nm and observed as the bandpass of red fluorescence filtered from 552 to 617 nm. We thus compared the number of dead cells in each group showing only blue fluorescence with SYTOX-blue. For analysis, we identified CA1 pyramidal cell layer before counting and normalized the data based on the total number of nuclei per mm CA1 region.

### Fluorometric Assessment of Cytosolic Ca^2+^ Changes

To evaluate changes in cytosolic Ca^2+^ concentrations, Fura2 (DOJINDO, Kumamoto, Japan) fluorescence signals of whole-cell voltage-clamped pyramidal cells were measured by adding 15 µmol/L of Fura2 to the internal pipette solution. Using a fast-switching multi-wavelength illumination system (Lambda DG-4; Sutter Instruments, CA, USA), Fura2 fluorescence signals were excited at 340 nm and 380 nm every 10 s and emission was filtered at 510 nm with a 500-nm dichroic mirror. To acquire these images, a × 40 water immersion objective lens (LUMPlanFI/IR; Olympus, Japan) and a CCD camera (ORCA-flash 4.0 v3; Hamamatsu Photonics, Shizuoka, Japan) were used. All images were saved in MetaMorph software (Molecular Devices, CA, USA) and for analyses, we measured a region of interest (ROI) defined as a circular area 5 µm in diameter with maximum fluorescence intensity near the center of the recording cell. Mean fluorescence intensity ratio for 340-nm and 380-nm excitations in the ROI was calculated.

### Fluorometric Assessment of Mitochondrial Membrane Potential

To evaluate changes in mitochondrial membrane potential during the anoxic and reperfusion periods, we used JC1 (Cayman Chemical, MI, USA) fluorescence, for which the emission wavelength changes depending on the mitochondrial membrane potential. JC1 was loaded into cytoplasm via patch pipette. The tip of the patch pipette was filled with an internal solution and from the back of the pipette an internal solution containing 2.0 µM of JC1 was added immediately before use. The red fluorescence of JC1 represents the J-aggregated state excited at 548 nm with a 580-nm dichroic mirror and fluoresced at 590 nm with a long-pass filter. The green fluorescence of JC1 represents the monomeric state excited at 477 nm with a 500-nm dichroic mirror and fluoresced with a bandpass filter at 515–565 nm. Fluorescence images were acquired every 30 s using the same apparatus used for Fura2. To measure fluorescence, the ROI was defined as a hand-drawn polygonal area covering the region of high red fluorescence, because the red fluorescence was eccentrically distributed around the nucleus and was often crescent shaped. The averages of green and red fluorescence in the ROI were calculated as green/red ratios for analysis. An increase in this green/red ratio indicates mitochondrial depolarization.

### Statistical Analysis

Each experimental value of cumulative sEPSC occurrence, NMDA amplitude, Fura2 340/380 ratio, and JC1 green/red ratio was calculated as a percentage relative to the mean value observed during the 5-min pre-anoxic period for each pyramidal cell under conditions of control, PostC, sham and PostC with Ru265 perfusion at 1 µM, 10 µM, and 50 µM, respectively. All data are expressed as mean ± standard deviation (SD) in the graph. For statistical analysis, the Shapiro–Wilk normality test was performed for experimental data in each group to check whether distributions were normal. When data were normally distributed, homogeneity of variance in these data was checked using Levene’s test. For multiple testing, we applied the Tukey method followed by Welch’s one-way analysis of variance. On the other hand, percentage data that were not normally distributed were analyzed using the Kruskal–Wallis test, followed by the Mann–Whitney U test for each of the two groups. Moreover, significant effects were further tested with a post-hoc multiple comparison test using the Steel–Dwass method. Significant differences were set at the level of *p < 0.05 and **p < 0.01. Sample size and power calculations based on data from our previous studies were calculated using the effect size of each experiment as about 27–84%, with power of 0.8 and α of 0.05 in each experiment. Protocols in all experiments were not performed in a randomized or blinded manner, so we planned to perform at least five experiments in each group to minimize subjective biases. R software and G power software were used for statistical analyses of all data.

## Results

### Changes to sEPSCs

**Fig. 2:**
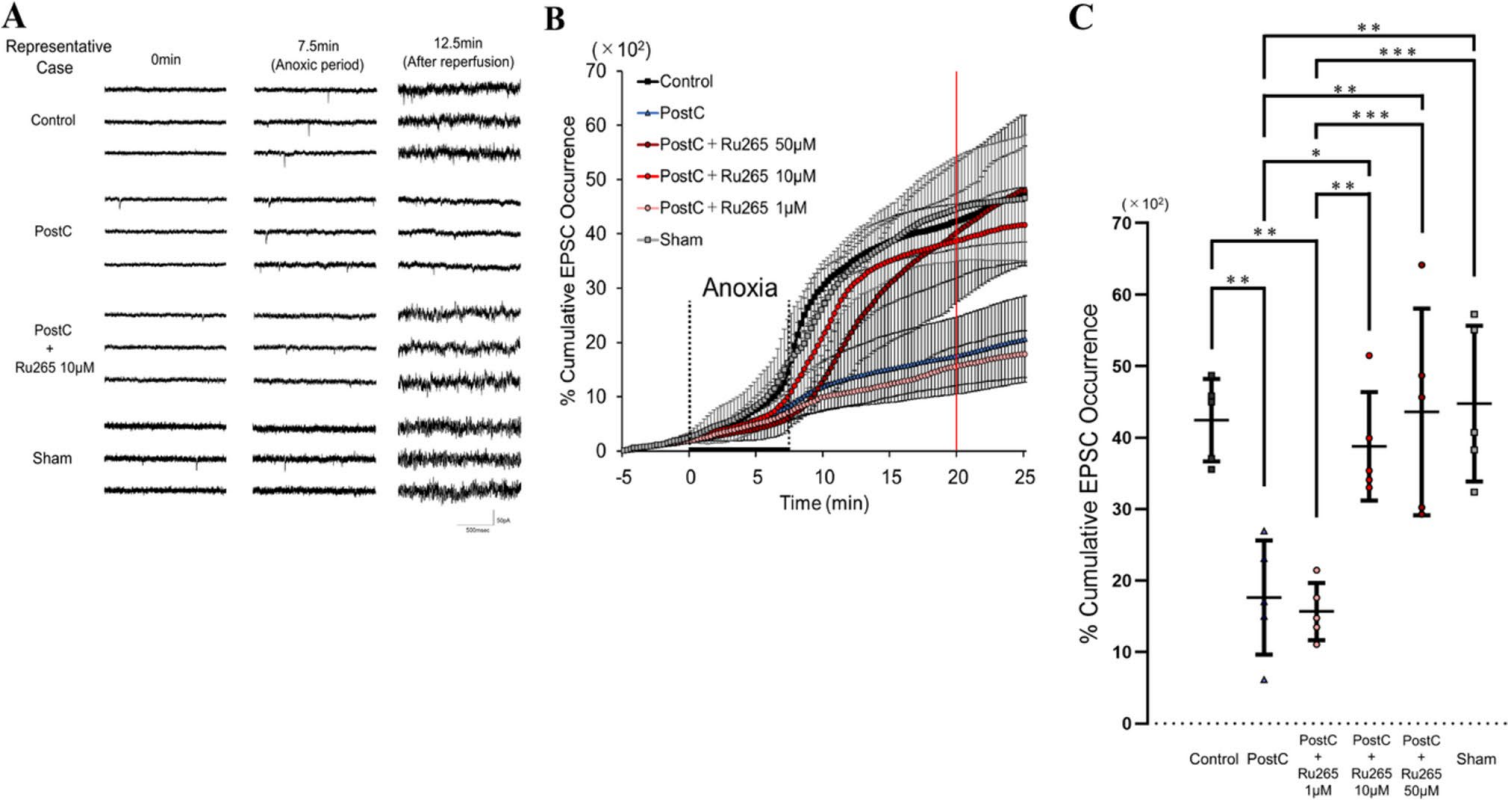
Changes to spontaneous excitatory post-synaptic currents (sEPSCs). **A** Representative case of sEPSCs for the control (top), PostC (upper-middle), PostC + Ru265 10 µM (lower-middle), and Sham (bottom) groups during pre-anoxia and anoxic and reperfusion periods. In each trace, sEPSCs caused by synaptic glutamate release were observed as transient downward deflections (inward currents). **B** Time course of cumulative sEPSC occurrence in the control, PostC, PostC + Ru265, and sham groups. In each group, markers and bars represent the mean and standard deviation (SD), respectively. Data on the red line were used for statistical analysis. **C** Comparison of mean and SD in each group. The sample size was calculated as at least *n* = 3 in each group. Each bar indicates the SD of cumulative sEPSC occurrence at 20 min. Asterisks indicate significant difference in Tukey’s multiple comparisons test (*p < 0.05, **p < 0.01, ***p < 0.001). *sEPSCs* spontaneous excitatory post-synaptic currents, *PostC* ischemic postconditioning, *Ru265* Ruthenium red 265; sham, control + Ru265 10 µM

Initially, we checked the occurrence of sEPSCs in each group to confirm whether adjustments to this interaction process of PostC could be made by adjusting MCUs with an MCU blocker. Representative data from the control, PostC, sham, and PostC + Ru265 10-µM groups are shown in Fig. 2A. In all groups, sEPSCs began to increase approximately 7 min after the anoxic period started, while in the PostC group alone, this increase in sEPSCs receded to pre-anoxia levels immediately after reperfusion. Conversely, an explosive increase in sEPSC frequency was seen 2 min after reperfusion in the control, sham, and PostC + Ru265 10 µM groups. According to our previous data, we calculated the effect size as *r* = 0.845 and estimated the sample size in each group for this experiment as *n* = 3 at least. In this experiment, cumulative data on sEPSC occurrence in the control, sham, PostC, and PostC + Ru265 groups included *n* = 5 data in each group. The time course of cumulative sEPSC occurrence in Fig. 2B shows that the frequency of sEPSCs gradually increased during the anoxic period in all groups. With reperfusion after 7.5 min of anoxic load, sEPSC frequency in the control, sham, and PostC + Ru265 10-or 50-µM groups was sharply increased. We used cumulative occurrence at 12.5 min after reperfusion for statistical analysis. These data were estimated as showing a normal distribution according to the Shapiro–Wilk test (Supplementary Table 1). The percentage of cumulative sEPSC occurrence was significantly higher in the control group than in the PostC or PostC + Ru265 1-µM groups (Fig. 2B, Supplementary Table 1). Moreover, the percentage was also significantly higher in the PostC + Ru265 10-µM group than in the PostC or PostC + Ru265 1-µM group (Fig. 2C, Supplementary Table 1). Similarly, the percentage in the PostC + Ru265 50 µM or sham group was significantly higher than that in the PostC or PostC + Ru265 1-µM group (Fig. 2B, C, Supplementary Table 1). Furthermore, to examine the optimal concentration of Ru265 in this experimental setting, we set three different concentrations of Ru265 administered in ACSF: low (1 µM); medium (10 µM); and high (50 µM). As mentioned above, effects of Ru265 on the suppression of sEPSCs by PostC were observed at 10 µM and 50 µM (Fig. 2B). We thus determined that Ru265 at 10 µM was the optimal concentration in this experiment.

### Inhibition of MCU by Ru265 Prevents Suppression of NMDAR Currents by PostC

**Fig. 3:**
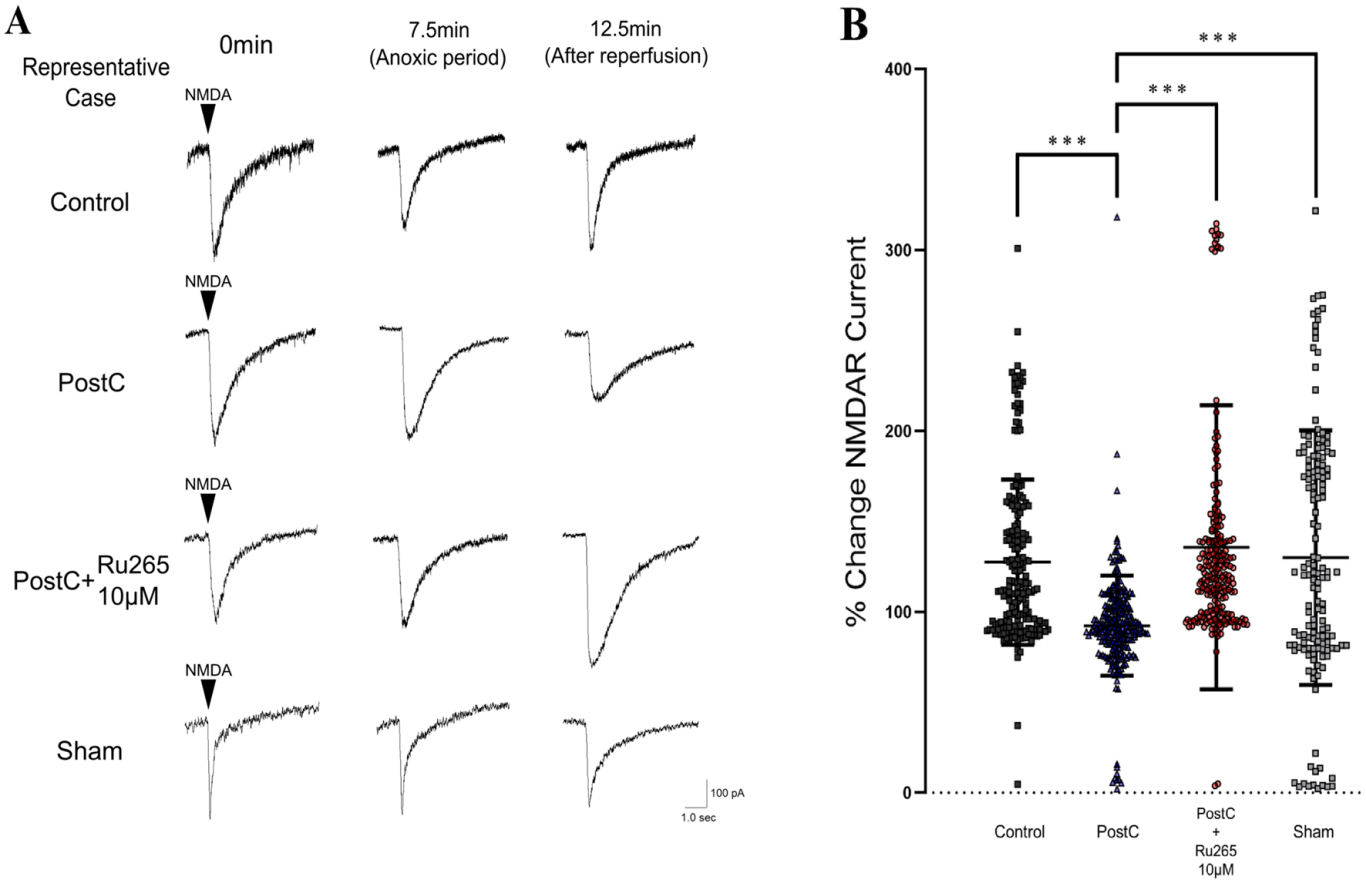
Recording of *N*-methyl-d-aspartate (NMDA)-induced currents. **A** Representative traces of NMDA receptor (NMDAR) currents during pre- and post-anoxic periods in each group. NMDAR current amplitude was observed as the wave with slow decay for several seconds. **B** Bar graph and plot shows changes in mean peak amplitude of NMDAR currents from 10 to 20 min after reperfusion and SD. Total sample size was calculated as at least *n* = 59. Asterisks indicate a significant difference in the Steel–Dwass multiple comparisons test (***p < 0.001). *NMDA* *N*-methyl-d-aspartate, *PostC* ischemic postconditioning, *Ru265* ruthenium red 265; sham, control + Ru265 10 µM

Our previous research reported that PostC induced an interaction between mitochondria and NMDA receptors. To check how inhibition of MCU changed and affected this interaction, we recorded NMDAR currents every 30 s in each group. When NMDA was locally applied to CA1 pyramidal cells, NMDAR currents with slow decay for several seconds were observed in all three groups. After reperfusion, NMDAR currents decreased in the PostC group, while in the control, sham, or PostC + Ru265 groups, no change or rather an increase in NMDAR currents was observed (Fig. 3A). For statistical analysis, we calculated the change in mean peak amplitude of NMDAR currents from 10 to 20 min in each group, with values expressed as the percentage relative to mean peak amplitude during 5-min normoxia before the anoxic period. We calculated the effect size as *r* = 0.434 based on our previous data and estimated the total sample size for this experiment as *n* = 59 at least. The result in Fig. 3B shows that the percentage of NMDAR current amplitude after reperfusion was significantly larger in the control group and sham group than in the PostC group (Supplementary Table 2). Similarly, in the PostC + Ru265 10-µM group, the percentage NMDAR current amplitude after reperfusion was significantly larger than that in the PostC group (Fig. 3B, Supplementary Table 2). These results therefore indicate that the inhibition of MCU by Ru265 prevented the suppressive effects of PostC on NMDAR currents.

### Inhibition of MCU by Ru265 in PostC Increases the Number of Dead CA1 Neurons

**Fig. 4:**
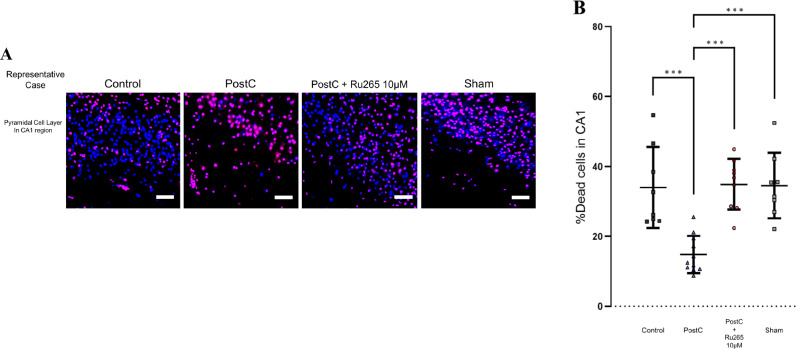
Dead cells count in the CA1 region. **A** Representative microscopic view in the CA1 region and nuclei of dead cells in each group. White scale bars, 50 µm. Cells that died due to I/R injury are observed as blue-colored CA1 neurons. **B** Data of neurons that died due to I/R injury per 1 mm of the CA1 region are plotted on each bar graph of mean and SD. Total sample size was calculated as at least *n* = 8. Asterisks indicate significant difference in Steel–Dwass multiple comparisons test (***p < 0.001). *PostC* ischemic postconditioning, *Ru265* ruthenium red 265; sham, control + Ru265 10 µM

Our previous study revealed that the number of dead CA1 neurons was reduced under the neuroprotection of PostC. We thus hypothesized that MCU inhibition might weaken the neuroprotection in PostC, leading to increased cell death after reperfusion. We therefore counted the number of dead CA1 neurons in situ for all four groups after reperfusion. To distinguish dead cells caused by I/R injury among all dead cells, we used two kinds of membrane-impermeable dye for nuclear staining at different wavelengths of fluorescence. In detail, dead cells due to the preparation procedure for hippocampal slices were observed as magenta cells stained with both PI and SYTOX-blue, while blue cells stained only with SYTOX-blue were considered as cells that died due to I/R injury. Thus, for statistical analysis, we counted the number of CA1 neurons stained blue as those that died during 20 min to 3 h of reperfusion after 7.5 min of anoxic insult (Fig. 4A). In this experiment, we calculated the effect size as *r *= 0.847 and estimated the total sample size for this experiment as at least n = 8. Numbers of dead CA1 neurons in the PostC group were significantly lower than in the control group and sham group (Fig. 4B, Supplementary Table 3). This result was similar to our previous study. Furthermore, significant differences were evident between the PostC and PostC + Ru265 10-µM groups (Supplementary Table 3). Dead CA1 neurons were thus significantly increased in the PostC + Ru265 and control groups compared to in the PostC group, indicating that inhibition of MCU weakened the neuroprotective effects against I/R injury from PostC and led to cell death. MCU was thus regarded as playing an important role in the neuroprotective effects of PostC.

### Ru265 Inhibits Suppression of Cytosolic Ca^2+^ Increase by PostC

**Fig. 5:**
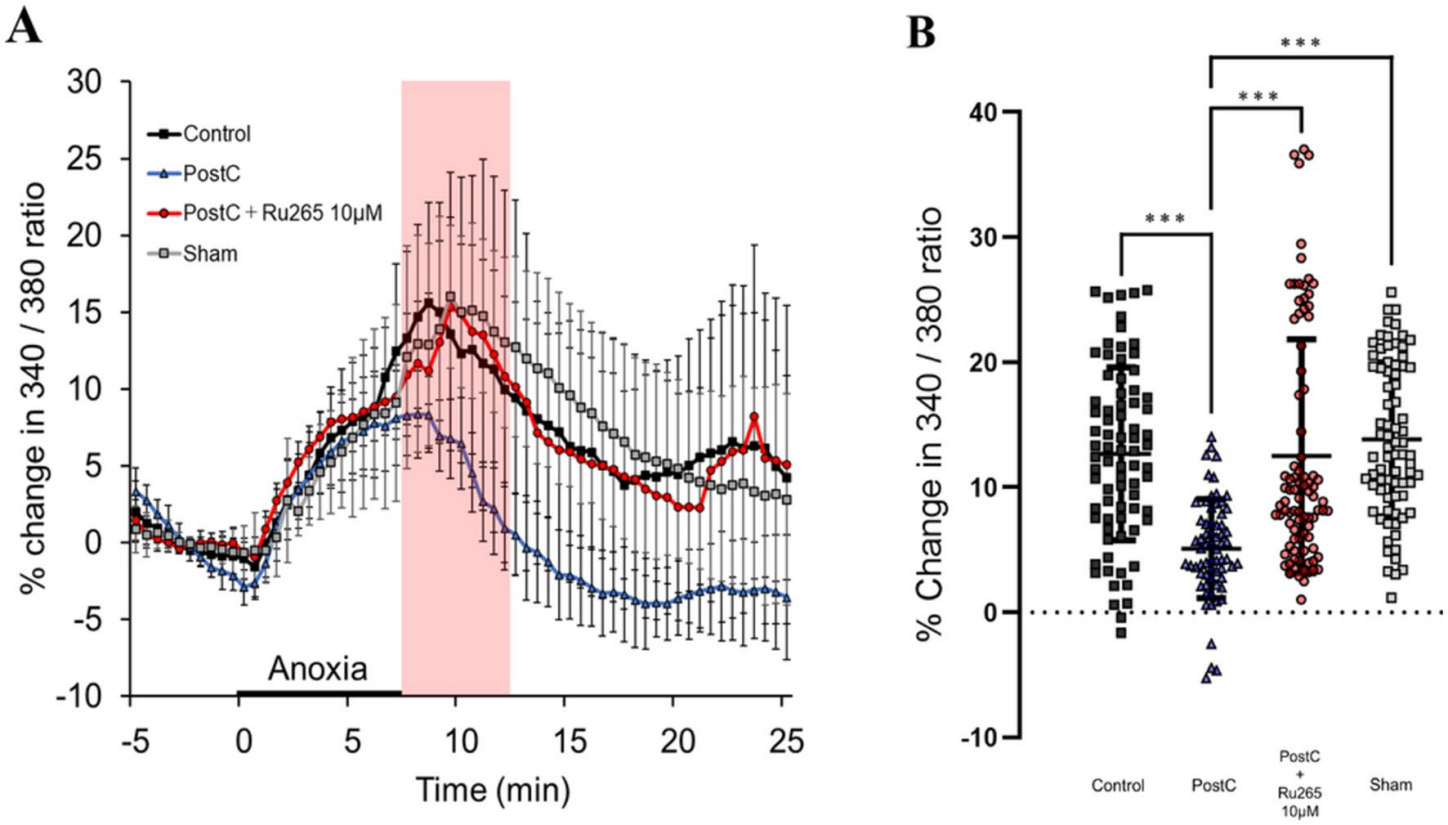
Changes in Fura2 ratio and cytosolic Ca^2+^ concentration. **A** Markers and bars represent mean values and standard deviations (SDs) during the time course of cytosolic Ca^2+^ concentration changes in Fura2. The pink band indicates the period from 0 to 5 min after the end of the anoxic period and was used for statistical analysis. **B** Bar graph and plot shows changes in Fura2 ratio during 0–5 min after 7.5 min of anoxia and the SD. Sample size was calculated as at least *n* = 41 in each group. Asterisks indicate significant difference in Steel–Dwass multiple comparisons test (***p < 0.001). *PostC* ischemic postconditioning, *Ru265* ruthenium red 265; sham, control + Ru265 10 µM

We examined the involvement of the MCU in changes to cytosolic [Ca^2+^] concentration by PostC. Change in the Fura2 ratio (340 nm/380 nm ratio) was calculated relative to the mean value observed during the 5-min pre-anoxic period. An elevation of this ratio indicated an increase in cytosolic Ca^2+^ concentration. In all groups, Fura2 ratio gradually increased during the anoxic period. After the end of the anoxic period, the ratio started to decrease gradually in the PostC group, but started to increase immediately in the control, sham, and PostC + Ru265 groups (Fig. 5A). The increase in intracellular Ca^2+^ concentration was thus suggested to be suppressed in the PostC group, with Ru265 weakening this effect. For this experiment, we calculated the effect size as *r* = 0.30 and estimated the sample size in each group as at least *n* = 41. We used the data of Fura2 ratio from 0 to 5 min after the anoxic period for statistical analysis. In the PostC + Ru265 10-µM group, the percentage change in Fura2 ratio was significantly higher than that in the PostC group, similar to that in the control group and sham group from 0 to 5 min after the anoxic period (Fig. 5B, Supplementary Table 4). This result suggests that suppression of cytosolic [Ca^2+^] increase in the early stage of reperfusion by PostC is prevented through the inhibition of MCU activity by Ru265.

### Ru265 Inhibits the Interaction Between Mitochondrial Depolarization by PostC and MCU

**Fig. 6:**
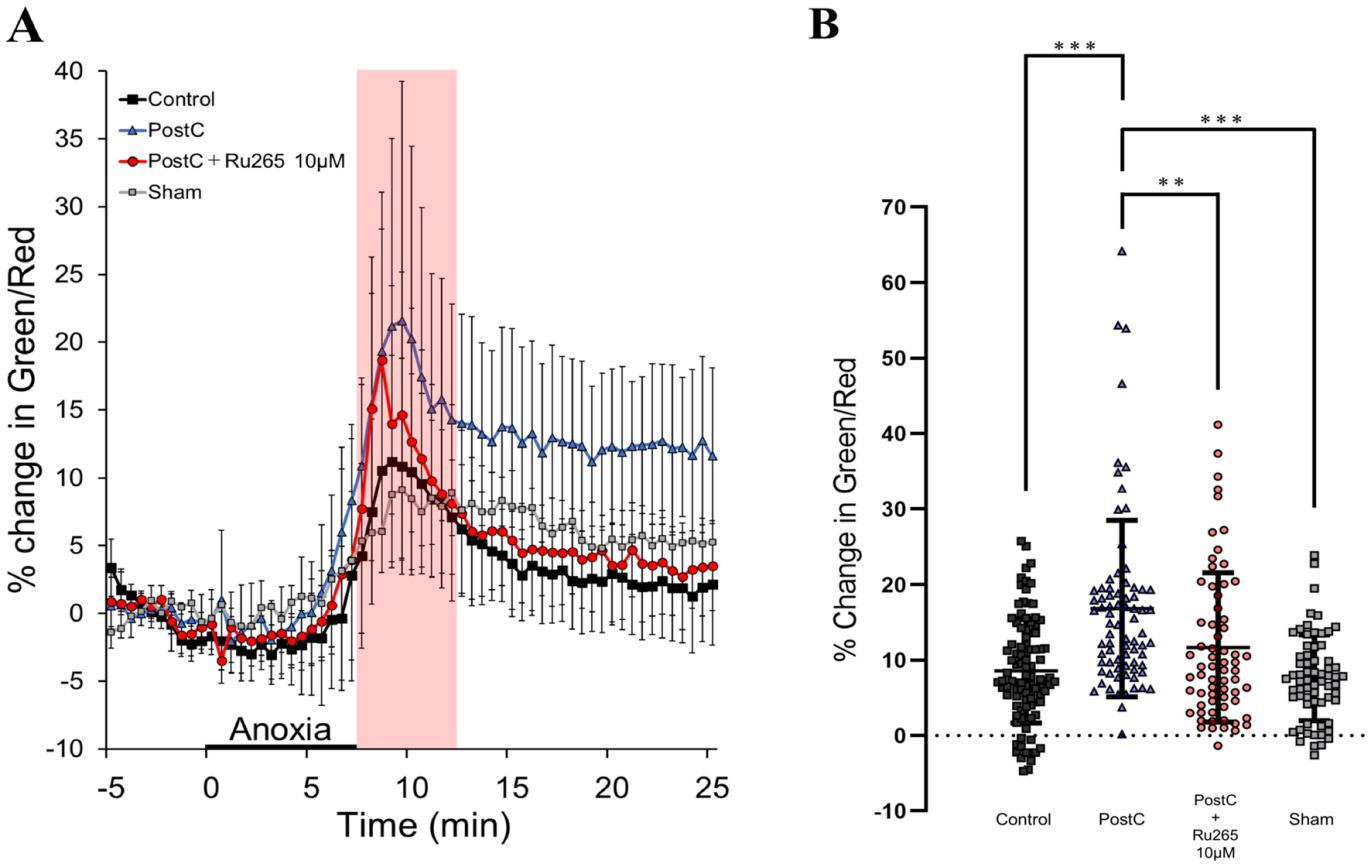
Investigation of mitochondrial membrane potential in each group. **A** Markers and bars represent mean values and standard deviation (SDs) during the time course of changes in mitochondrial membrane potential by JC1. The pink band indicates the period from 0 to 5 min after the end of the anoxic period and was used for statistical analysis. **B** Bar graph and plot shows changes in JC1 ratio during 0–5 min after 7.5 min of anoxia and the SD. Sample size was calculated as at least *n* = 49.5 in each group. Asterisks indicate significant difference in Steel–Dwass multiple comparisons test (*p < 0.05, ***p < 0.001). *PostC* ischemic postconditioning, *Ru265* ruthenium red 265; sham, control + Ru265 10 µM

In our previous study, mitochondrial depolarization appeared to be a key factor in the neuroprotection provided by PostC. Thus, to examine whether MCU mediates the depolarization of the mitochondrial membrane induced by PostC, we investigated mitochondrial membrane potential with JC1 fluorescence. An increase in JC1 green/red ratio indicates depolarization of the mitochondrial membrane, with percentages calculated relative to the mean value observed during the 5-min pre-anoxic period. After the anoxic period at 7.5 min, the ratio immediately increased in the PostC and PostC + Ru265 groups. Subsequently, the ratio started to decrease in the PostC + Ru265 group but not in the PostC group (Fig. 6A). Changes in green/red ratio from 0 min until 5 min after the end of the anoxic period were used for analysis. In this experiment, we calculated the effect size as *r* = 0.27 based on our previous similar study and estimated the sample size in each group as at least *n* = 49.5. The result from Fig. 6B showed that the green/red ratio in the early phase from 0 to 5 min after the end of the anoxic period was differed significantly between the PostC group and the PostC + Ru265 10-µM group, which was similar to the control and sham group (Supplementary Table 5). These results suggest that in the PostC + Ru265 group, mitochondrial depolarization by PostC is weakened after reperfusion.

## Discussion

This study demonstrates the role of MCU in the mechanisms underlying PostC protection against I/R injury, using the whole-cell patch-clamp technique on mouse hippocampal CA1 pyramidal cells. Blocking MCU with Ru265 weakened the effect of PostC. As a result, the number of dead cells in the CA1 region increased because inhibition of MCU weakened the effect of reducing Ca^2+^ influx into the cytosol via NMDAR by PostC. Moreover, in PostC with an inhibitor of MCU, depolarization of the mitochondrial membrane was elevated in the early phase after reperfusion, but weakened immediately. These results suggest that interactions between mitochondrial depolarization and MCU activation are involved in the PostC mechanism, leading to neuroprotection by reducing NMDAR currents and Ca^2+^ influx into the cytosol.

### Regulation of MCU Affects the PostC Pathway and Reduces Neuroprotective Effects in PostC After Reperfusion

In our previous research, we reported that PostC induces interactions between mitochondria and NMDAR, and that the observed increase in sEPSCs was an indirect result of this interaction (Morisaki et al. 2022; Yokoyama et al. 2019). In this study, we initially checked the occurrence of sEPSCs in each group to confirm whether adjustments to this interaction process in PostC could be made by adjusting the MCU with MCU blockers. The occurrence of sEPSCs in the PostC + Ru265 group was sharply increased, compared to the PostC group, as shown in Fig. 2. This result suggests that MCU plays a key role in the PostC pathway. During I/R injury, an excessive release of glutamate occurs, leading to over-activation of NMDAR (Bonova et al. 2013; Dávalos et al. 1997; Soria et al. 2014). This cascade leads to cell necrosis or apoptosis by excess Ca^2+^ influx into the neuron, triggering a range of downstream pro-death signaling events such as calpain activation, generation of reactive oxygen species and damage to mitochondria (Curcio et al. 2016; Kristián and Siesjö 1998; Lau and Tymianski 2010). Yin et al. suggested that NMDAR mediates PostC-induced neuroprotection (Yin et al. 2005) and Morisaki et al. reported that low-conductance opening of mPTP by PostC induces extrusion of small amounts of Ca^2+^ into the cell cytosol, so some chemical mediators were predicted to inhibit NMDAR over-activation against excess Ca^2+^ influx into neurons (Morisaki et al. 2022). Moreover, in their experiments, increased intracellular Ca^2+^ concentrations after reperfusion were not observed in either control or PostC group when the extracellular Ca^2+^ concentration was set to 0. From this result, they concluded that in PostC, suppression of NMDAR over-activation during reperfusion inhibited Ca^2+^ influx into the cytoplasm from the extracellular space, reducing Ca^2+^ overload in the cytoplasm and cellular damage (Morisaki et al. 2022). Therefore, death signaling from NMDAR over-activation is considered the key to the neuroprotective mechanisms of PostC. The present study showed that cytosolic Ca^2+^ concentrations during the anoxic-to-reperfusion period increased sharply due to inhibition of the MCU by Ru265, similar to that observed in the control group, while no such changes were observed in PostC after reperfusion (Fig. 5). In addition, the reduction of NMDAR currents was also weakened by inhibiting the MCU (Fig. 3). Thus, our results also suggest that the inhibition of MCU-mediated Ca^2+^ influx into the mitochondria disrupts the PostC process, resulting in a loss of PostC-induced neuroprotection against extracellular Ca^2+^ influx through NMDAR over-activation.

Under physiological conditions, cytosolic Ca^2+^ concentrations are kept at around 100 nM by intracellular Ca^2+^ stores, Ca^2+^ channels and pumps in the plasma membrane and organelle membrane, and Ca^2+^-binding proteins (Babcock et al. 1997; Berridge 2016; Carvalho et al. 2020; Chen et al. 2020; Enomoto et al. 2017). The endoplasmic reticulum is known as the largest intracellular Ca^2+^ store, but mitochondria, lysosomes, and the nucleus are also involved in regulating intracellular Ca^2+^ concentrations (Kaufman and Malhotra 2014; Smaili et al. 2013; Samanta and Parekh 2017). However, the influx of cytosolic Ca^2+^ into the mitochondrial matrix via the MCU, in particular, leads to elevated mitochondrial Ca^2+^ concentrations and influences various mitochondrial metabolic processes, including mitochondrial respiration, ATP production, mitophagy/autophagy, and even the death pathways of apoptosis or necrosis (Duchen 1999; East and Campanella 2013; Gottlieb and Bernstein 2016). Furthermore, particularly under ischemic conditions, anaerobic glycolysis in cells starts to work as the primary metabolic pathway, leading to an increase in lactic acids and a decrease in cytosolic pH. While cytosolic pH decreases, during ischemia the mitochondrial membrane potential is diminished and, in case of glial cells, glutamate increases the mitochondrial respiration rate and pH-gradient of mitochondrial matrix and leads to the supportive effect to oxidative phosphorylation (Krasil’nikova et al. 2019; Surin et al. 2022). After reperfusion, the rapid restoration of the mitochondrial membrane potential with the return of oxygen into the cytosol provides a strong driving force for the entry of cytosolic Ca^2+^ into the mitochondrion via the MCU (Shintani-Ishida et al. 2012). This results in mitochondrial Ca^2+^ overload, leading to cell damage or death associated with opening of the mPTP. This process generates toxic products such as cytochrome C and swelling of the matrix, eventually causing outer mitochondrial membrane rupture (Bonora et al. 2017; Hawrysh and Buck 2013; Morciano et al. 2015; Shintani-Ishida et al. 2012). Thus, some reports have stated that inhibition of the MCU reduces the cell damage caused by mitochondrial Ca^2+^ overload and may confer neuroprotection (Novorolsky et al. 2020; Woods and Wilson 2019; Zhao et al. 2015). However, our results (Fig. 4) initially appear to contradict this contention, showing an increase in dead cells in the CA1 region after inhibition of the MCU in PostC. Nonetheless, inhibition of the MCU cannot prevent other toxicity caused by cytosolic Ca^2+^ overload (Curcio et al. 2016; Kristián and Siesjö 1998; Lau and Tymianski 2010). Thus, the reason dead cells in the CA1 region are increased with MCU inhibition in PostC may be that other death signals related to cytosolic Ca^2+^ overload, not simply mitochondrial Ca^2+^ overload via the MCU, were activated and led to cell death.

### MCU Plays a Role as a Key Factor in the Interactive Processes of Mitochondrial Depolarization in PostC

In our previous research, we reported that opening of the mito-K_ATP_ channel triggered the PostC mechanism and caused the mitochondrial depolarization observed during the early period of reperfusion in the PostC group, but not in the control group (Morisaki et al. 2022). Moreover, we also reported that a key factor in the neuroprotection conferred by PostC was the low-conductance opening of the mPTP. The catastrophic process of cell injury is induced through the high-conductance mode of mPTP opening, which allows the passage of ions, including Ca^2+^, leading to dissipation of the mitochondrial membrane potential and eventually resulting in cell death (Brenner and Moulin 2012). Conversely, under physiological conditions, the mPTP could exhibit intermittent opening in low-conductance mode, contributing to intracellular Ca^2+^ homeostasis and the regulation of mitochondrial function (Giorgio et al. 2013). We also reported that the low-conductance mode of mPTP opening prevented opening in high-conductance mode and reduced NMDAR conductance (Morisaki et al. 2022). Furthermore, mitochondrial membrane potential switches between these two modes, with the threshold value controlled by mitochondrial Ca^2+^ concentration (Bazil et al. 2010). Opening of the mito-K_ATP_ channel is triggered by a local decrease in cytosolic ATP under ischemic stress, subsequently leading to increased K^+^ influx into the mitochondrial matrix (Hawrysh and Buck 2013; Pamenter et al. 2008). In PostC, opening of this channel leads to mitochondrial depolarization, reducing the driving force of Ca^2+^ influx into the mitochondrial matrix and abrogating excessive Ca^2+^ accumulation in the matrix, thus avoiding the high-conductance mode of mPTP opening (Hawrysh and Buck 2013; Morisaki et al. 2022). Mitochondrial depolarization is thus another key factor in the neuroprotection provided by PostC. In the present study, depolarization of the mitochondrial membrane was weakened after reperfusion in the PostC + Ru265 group, compared to in the PostC group. Such data suggest that in PostC, mitochondrial depolarization is triggered by opening of the mito-K_ATP_ channel, but some form of interaction between the mito-K_ATP_ channel and MCU was blocked by Ru265, resulting in a loss of neuroprotective effects through mitochondrial membrane potential in PostC. Furthermore, our findings also suggest that mild interactive regulation of the MCU provided by the driving force from the mito-K_ATP_ channel is necessary for maintaining mitochondrial depolarization in PostC. Thus, we consider that it is necessary to investigate more specifically how the mitochondrial metabolism in PostC is regulated with further research about, such as the mitochondrial Ca^2+^ level and the mitochondrial respiration in future.

### Pharmacological Approach to the MCU

The present study used ruthenium red 265 as a novel selective inhibitor of the MCU. The most well-known and commonly used MCU inhibitor is Ru360, named for its strong absorbance at 360 nm (Emerson et al. 1993). However, Ru360 is poorly permeable and unstable in aqueous solution, losing activity within days (Hajnóczky et al. 2006; Márta et al. 2021). On the other hand, Novorolsky et al. reported that in a lysate of HEK293 or Hela cells, Ru265 was taken into these cells at 2–10 times greater rates than other structural analogs of ruthenium after these cells were incubated with each ruthenium complex at 50 µM for 24 h; moreover, the rise in mitochondrial Ca^2+^ concentration was observed rapidly, after only 30 min of incubation (Novorolsky et al. 2020; Woods et al. 2019). With our PostC protocol in previous research, we observed each single-cell action during a very short time course, including normoxia and anoxia within about 30 min to 1 h. Use of a rapidly permeable drug was thus necessary for this protocol, and from this point of view, we regarded Ru265 as suitable for our research (Novorolsky et al. 2020). Furthermore, ruthenium compounds are also generally known for their cytotoxicity (Alessio 2017; Dutta et al. 2008; Peacock et al. 2006; Hartinger et al. 2008; Lameijer et al. 2017; Mühlgassner et al. 2012; Süss-Fink 2010; Wachter et al. 2012; Wang et al. 2003; Wee and Dyson 2006), while Ru265 is a less toxic compound without observable effects on mitochondrial membrane potential or other intracellular Ca^2+^ dynamics reportedly (Woods et al. 2019). Thus, in our present study, it was also observed that Ru265 itself did induce no change to all the parameters such as the EPSC occurrence, NMDAR currents, intracellular Ca^2+^ concentration and mitochondrial depolarization in the sham (Control + Ru265 10 µM) group, similar to the control group. These results suggest that Ru265 had less affection or toxicity to cell membranes or some other cell organelles.

### Limitations and Future Direction

In our previous and present studies, we used the hippocampal pyramidal cell of male mice only. Recently in the clinical and experimental studies, it is discussed about the gender-dependent difference of the brain injury. They reported that the male neonates were more susceptible to the damages related to brain ischemia, resulting in more severe neurological outcome compared to females (Cikla et al. 2016; Hill and Fitch 2012; Uluç et al. 2013). Thus, our results might be different if we had used female mice and for the future prospection, we should consider about the ‘gender specificity in PostC.’

In our laboratory, ‘Safe pharmacological PostC’ represents our central concern for achieving clinical application. Diazoxide as the opener of the mito-K_ATP_ channel is unsuitable because of its toxic side effects (Kumar et al. 1976), while in our previous study, Furuta et al. reported on the neuroprotective mechanisms of melatonin-induced pharmacological PostC (Furuta et al. 2022) and melatonin could be a new candidate for pharmacological PostC without side effects. Thus, as future directions, in vitro and in vivo studies are needed to explore methods of delivering melatonin to the brain, such as catheterization of middle cerebral artery occlusion models. In addition, it is also necessary to find other PostC-like neuroprotective and less toxicity chemical compounds or drugs.

## Conclusion

The MCU plays an important role related to depolarization of the mitochondrial membrane in PostC, leading to reduced numbers of dead cells in the CA1 region through downregulation of the occurrence of NMDAR currents and reduced Ca^2+^ influx into cytosol.

## Supplementary Information

Below is the link to the electronic supplementary material.

Supplementary file1 (DOCX 1708 KB)

## Data Availability

The datasets of the current study are available upon request with no restriction.
