# Supplementary figures and images for: Mitochondrial Calcium Uniporter (MCU) is Involved in an Ischemic Postconditioning Effect Against Ischemic Reperfusion Brain Injury in Mice
Source: Cell Mol Neurobiol. 2024 Apr 3;44:32. doi: 10.1007/s10571-024-01464-7 (PMC10991049; doi:10.1007/s10571-024-01464-7)

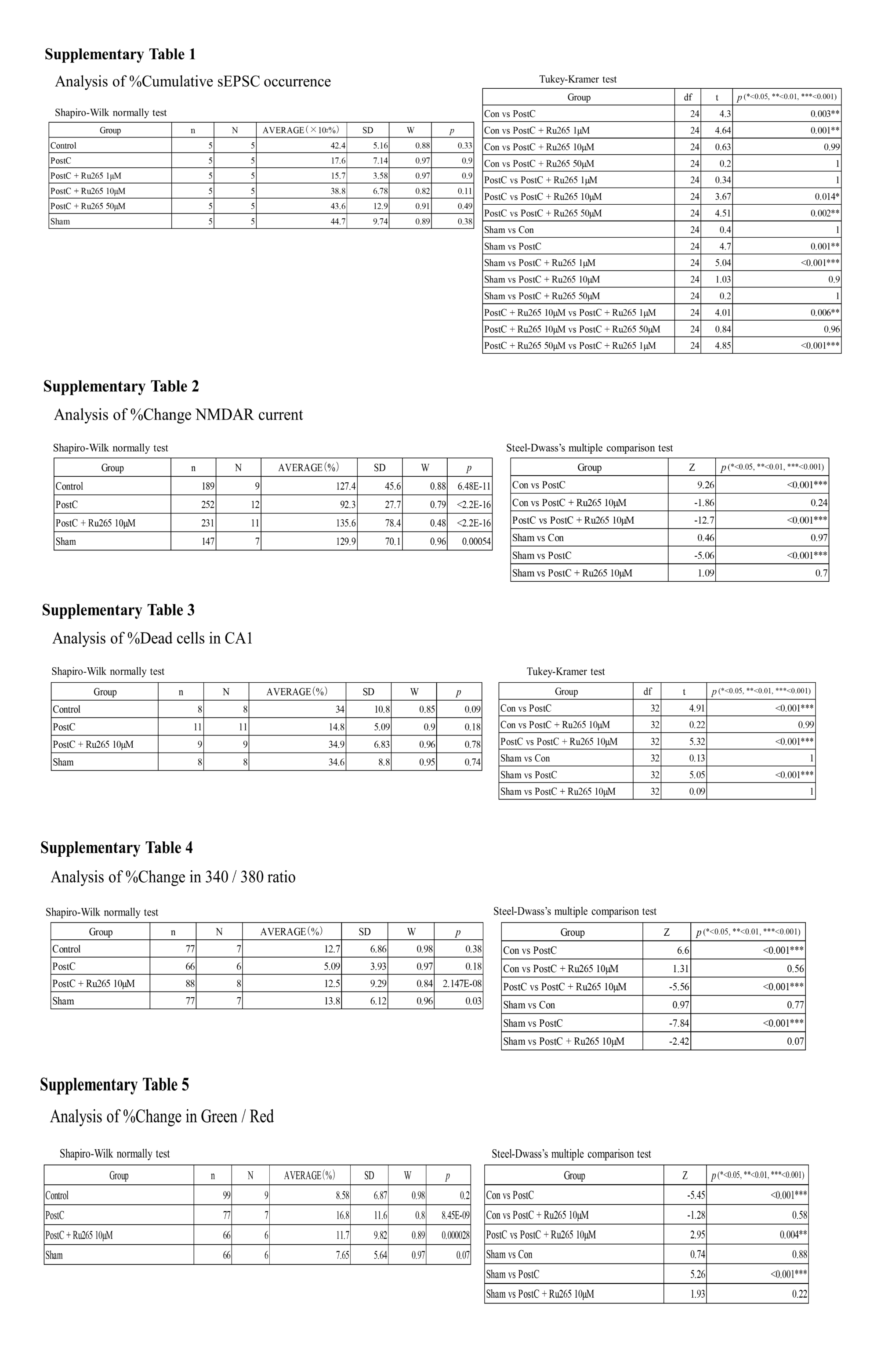

Supplement: Supplementary file 1 — Supplementary file1 (DOCX 1708 KB) [file 10571_2024_1464_MOESM1_ESM.docx]
